# Evidence of Convergent Evolution in Humans and Macaques Supports an Adaptive Role for Copy Number Variation of the β-Defensin-2 Gene

**DOI:** 10.1093/gbe/evu236

**Published:** 2014-10-27

**Authors:** Barbara Ottolini, Michael J. Hornsby, Razan Abujaber, Jacqueline A.L. MacArthur, Richard M. Badge, Trude Schwarzacher, Donna G. Albertson, Charles L. Bevins, Jay V. Solnick, Edward J. Hollox

**Affiliations:** ^1^Department of Genetics, University of Leicester, United Kingdom; ^2^Department of Microbiology and Immunology, University of California Davis School of Medicine; ^3^Helen Diller Family Comprehensive Cancer Center, University of California San Francisco; ^4^Department of Biology, University of Leicester, United Kingdom; ^5^Department of Medicine, Center for Comparative Medicine, and the California National Primate Research Center, University of California; ^6^Present address: European Molecular Biology Laboratory, European Bioinformatics Institute, Wellcome Trust Genome Campus, Hinxton, Cambridge, United Kingdom; ^7^Present address: Bluestone Center for Clinical Research, New York University College of Dentistry, New York, New York

**Keywords:** defensin, copy number variation, macaque, genome structure, evolution

## Abstract

β-defensins are a family of important peptides of innate immunity, involved in host defense, immunomodulation, reproduction, and pigmentation. Genes encoding β-defensins show evidence of birth-and-death evolution, adaptation by amino acid sequence changes, and extensive copy number variation (CNV) within humans and other species. The role of CNV in the adaptation of β-defensins to new functions remains unclear, as does the adaptive role of CNV in general. Here, we fine-map CNV of a cluster of β-defensins in humans and rhesus macaques. Remarkably, we found that the structure of the CNV is different between primates, with distinct mutational origins and CNV boundaries defined by retroviral long terminal repeat elements. Although the human β-defensin CNV region is 322 kb and encompasses several genes, including β-defensins, a long noncoding RNA gene, and testes-specific zinc-finger transcription factors, the orthologous region in the rhesus macaque shows CNV of a 20-kb region, containing only a single gene, the ortholog of the human β-defensin-2 gene. Despite its independent origins, the range of gene copy numbers in the rhesus macaque is similar to humans. In addition, the rhesus macaque gene has been subject to divergent positive selection at the amino acid level following its initial duplication event between 3 and 9.5 Ma, suggesting adaptation of this gene as the macaque successfully colonized novel environments outside Africa. Therefore, the molecular phenotype of β-defensin-2 CNV has undergone convergent evolution, and this gene shows evidence of adaptation at the amino acid level in rhesus macaques.

## Introduction

Despite multiallelic copy number variation (CNV) being an established and important aspect of genetic variation, little is known about its evolution and population genetics, particularly when compared with the extensive experimental and theoretical body of work concerning single nucleotide variation (Schrider and Hahn 2010). In principle, multiallelic CNV is subject to the same population genetic forces as other variation, being generated by mutation, maintained by balancing selection or removed by directional selection or genetic drift. Because of genetic drift, most polymorphisms are transient and are very unlikely to be maintained during speciation and subsequent divergence. However, there is extensive evidence that CNVs can be shared across species boundaries (Bailey and Eichler 2006). In perhaps the best established example, CNV of β-defensin genes has been observed not only in humans and other great apes (Hollox et al. 2003; Hardwick et al. 2011; Sudmant et al. 2013) but also in dogs (Leonard et al. 2012), cattle (Bickhart et al. 2012), and pigs (Chen et al. 2012; Wang et al. 2013). Furthermore, the structurally similar crotamine gene shows CNV in *Crotalus durissus* rattlesnakes (Oguiura et al. 2009; Yount et al. 2009).

An explanation for the prevalence of β-defensin CNV across mammals might be that certain aspects of genome structure, such as regions rich in segmental duplications and high-copy number repeats (such as retroviral elements), occurred in an ancestor, were maintained across species boundaries, and sponsor recurrent CNV in different species lineages (Marques-Bonet, Girirajan, et al. 2009; Marques-Bonet, Kidd, et al. 2009; Gokcumen et al. 2013). Indeed, a notable case is the observation of CNV hotspots in great apes (Marques-Bonet, Kidd, et al. 2009). These are caused by inheritance of segmental-duplication-rich regions generated in a great ape ancestor along different lineages to different great ape species and therefore sponsoring likely recurrent CNVs in the same region in different species. These CNVs may or may not have a selective advantage, and given that complex genomic architecture generating CNV can be maintained by drift, the null hypothesis must be that CNV is selectively neutral (Lynch 2007; Young et al. 2008). However, in *Drosophila melanogaster* there is evidence for natural selection affecting CNV genomewide (Emerson et al. 2008) and CNV hotspots shared between humans, chimpanzees, and macaques are enriched for regions predicted to be functionally relevant, which has been interpreted as evidence for positive selection of these CNV hotspots (Gokcumen et al. 2011).

We reasoned that if the same genes were CNV in different species as a result of different distinct genomic events, of different sizes and sponsored by very distinct mutational events, then this would suggest convergent evolution and a possible adaptive explanation for the recurrent observation of CNV. Indeed, such convergent evolution of single nucleotide variation, where different mutations have resulted in a similar molecular or physiological phenotype, has supported well-defined cases of positive natural selection in humans (Ingram et al. 2009; Huerta-Sanchez et al. 2013).

Detailed characterization of CNV in different species is hampered by low resolution array comparative genomic hybridization (aCGH) data and poorly assembled genomes, particularly in regions that show CNV (Eichler et al. 2007). Here, we use tiling-resolution aCGH together with physical mapping and two complementary CNV typing methods to compare the nature and extent of β-defensin CNV in the macaque with that in humans. As well as being a model locus for complex multiallelic CNV, the evolution of β-defensins is interesting and important because of their function. β-defensins are small cationic peptides with a canonical six-cysteine motif that forms three disulphide bridges in a characteristic arrangement. They are potent antimicrobial peptides (Lehrer and Ganz 2002; Ganz 2003), and also have an immunomodulatory role, involved in signaling to cells mediating immune responses (Klotman and Chang 2006; Semple and Dorin 2012). In addition, some β-defensins have evolved to have a role in reproduction (Tollner et al. 2008; Zhou et al. 2013), pigmentation (Candille et al. 2007), and venom toxicity (Torres et al. 2000; Whittington et al. 2008).

It is likely that β-defensins have evolved in vertebrates through a birth-and-death process, with some β-defensins having clear orthologs across mammals yet others unique to particular clades, having been generated by recent duplication events (Maxwell et al. 2003). In humans, a cluster of seven very distinct β-defensins is within two repeat-rich regions on chromosome 8p23.1 termed REPP (for repeat-proximal) and REPD (for repeat-distal) (Giglio et al. 2001; Hollox et al. 2008). The cluster of β-defensins varies as a block, that is, it is one contiguous CNV not a region where several overlapping CNVs are observed. The β-defensin cluster shows both copy number polymorphism, commonly between two and seven copies per diploid genome, and positional polymorphism, whereby the cluster of β-defensins maps to REPD and polymorphically to REPP as well (Hollox et al. 2003; Abu Bakar et al. 2009). Clear orthologs for these seven genes can be identified across primates and show no evidence of positive selection on amino acid sequence within primates (Hollox and Armour 2008). However, in more distantly related mammals, for example rodents, their orthologs can be more difficult to define.

Here, we compare the human β-defensin CNV region with the orthologous rhesus macaque (*Macaca mulatta*) region on chromosome 8p23.1. The rhesus macaque lineage diverged from the human lineage around 25 Ma, and the rhesus macaque is a biomedical model organism with a sequenced genome (Rhesus Macaque Genome Sequencing Analysis Consortium et al. 2007). It is a successful species, having the widest geographical range of any nonhuman primate (Southwick et al. 1996). Macaque β-defensins are understudied, with the notable exception of DEFB126 which is a major component of the sperm glycocalyx (Tollner et al. 2008). Only one study has tested the antimicrobial effect of macaque β-defensins, focusing just on the human β-defensin 2 (hBD2) ortholog in *Macaca fascicularis*, and showing that the macaque ortholog has very different antimicrobial activity to the human hBD2 (Antcheva et al. 2004).

## Materials and Methods

### Ethics Statement

Macaques were housed at the California National Primate Research Center (CNPRC), which is accredited by the Association for the Assessment and Accreditation of the Laboratory Animal Care, International. All animal procedures were approved by the Research Advisory Committee of the CNPRC and the Institutional Animal Care and Use Committee at the University of California (Protocol number 177160). All possible efforts were made to minimize suffering in accordance with the recommendations of the National Research Council (Institute for Laboratory Animal Research 2010). Animals were cohoused in suspended stainless steel cages in an environment-controlled facility with an ambient temperature of 21–25 °C, a relative humidity of 40–60%, and a 12-h light/dark cycle. Water and commercial monkey chow were provided ad libitum and fresh fruit was provided twice weekly, with forage enrichment provided daily. Venesection was performed under ketamine anesthesia followed by the administration of analgesics to minimize discomfort. No animals were euthanized.

### DNA Samples

For macaques, DNA was isolated using standard techniques from peripheral whole blood, collected by venesection. Human DNA samples derived from immortalized lymphoblastoid cell lines were purchased from Coriell Cell Repositories (for the HapMap Collection) or from the European Collection of Cell Cultures hosted by Public Health England, Porton, Wiltshire (for HRC-1 human random control panel).

### Human Array

A custom NimbleGen tiling oligonucleotide array (NimbleGen Systems Inc.) was designed with 190,240 probes covering the defensin region (chr8:6185000-84681910; hg18). Probe design, array fabrication, and array CGH experiments, including DNA labeling, hybridization, array scanning, data normalization, and log_2_ copy-number ratio calculation were performed by NimbleGen Systems Inc. Array CGH was carried out on 68 cell line DNA samples with cell line AF0105 (Hollox et al. 2005) used as reference DNA for all hybridizations. Array data were analyzed using the SignalMap Software (NimbleGen Systems Inc.).

### Macaque Array

DNA was hybridized to Agilent Custom CGH 8 × 15 K oligo microarrays using Agilent Oligo aCGH Hybridization Kit, according to the manufacturer’s protocol. 155 probes corresponding to the rhesus macaque *TP53* region (chr16:7398881–7417940; rheMac2) and *NFKB1* region (chr5:95515208–95571818) were included on the array as negative control probes targeting non-CNV regions, and 312 probes were included targeting chromosome X, as positive control probes. Arrays were washed and scanned on an Agilent Microarray Scanner, and images processed using Agilent Feature Extraction Software v9.5.3. As each probe was repeated five times on the array, analysis was performed on the averaged log signal intensity ratio value for each data point. After normalization, each data set was analyzed for CNV boundary detection using BreakPtr, a computational approach for fine-mapping CNVs based on high-resolution CGH data (Korbel et al. 2007). A discrete-valued, bivariate hidden Markov model was generated using a provided training template based on results from human known large deletions and duplications, with a conservative transition probability of 1 × 10^−^^6^.

### Real-Time Quantitative Polymerase Chain Reaction

Real-time quantitative polymerase chain reaction (PCR) was carried out as previously described (Hornsby 2011). Briefly, we designed test primer pairs of matched product size amplifying *DEFB103* (5′-TATTATTGCAGAGTCAGAGGTGGCCG-3′ and 5′-GTGTCGAGCACTTGCCAATCTGTT-3′) and *DEFB2L* (5′-TACTGCTGCAGACACTCTGCCC-3′ and 5′-TGAACCTGCGGTGGCCTCGT-3′). A primer pair for β-globin, to act as a diploid copy number control, was also designed (5′-AAGTGGTGGCTGGTGTGGCTAATG-3′ and 5′-GGAACCTTTGGTAGAAATTGGACAGC-3′). Genomic DNA (10 ng) was amplified in 1 × SYBR green PCR Master Mix (Applied Biosystems) with 0.2 µM each of primer. Reactions were cycled for 5 min at 95 °C followed by 2 s at 95 °C, 5 s at 58 °C, and 20 s at 72 °C for 35 cycles. A standard curve was generated for each amplification using plasmids containing the concatenated test sequence and the reference sequence, and estimates of diploid copy number were made by comparing test and reference amplification dCT values during real-time PCR amplification, monitored by SYBR Green intercalating dye.

### Paralog Ratio Test

We designed six candidate primer pairs for paralog ratio test (PRT) to measure CNV of *DEFB2L* (Veal et al. 2013). Of the six, one pair of primers (5′-[FAM]GCCCTTTGAGCTGAGGCT-3′ and 5′-GGCCTAGGAGGAAAGAATGG-3′) amplified two specific correctly sized PCR products corresponding to the test locus (chr8:8075316–8075502) and the reference locus (chr14:70588986–70589098). The reference locus was not within a region of common CNV, according to previously published aCGH data (Lee et al. 2008). Genomic DNA (10 ng) was amplified in 10 µl of 45 mM Tris–HCI (pH 8.8), 11 mM NH_2_SO_4_ 4.5 mM MgCl_2_, 8.7 mM 2-mercaptoethanol, 4.5 µM ethylenediaminetetraacetic acid (EDTA), 1 mM of each dNTP, 110 µg/ml bovine serum albumin (Ambion), plus 0.5 µM each of primer, and 0.63 U of Taq DNA polymerase (Kapa Biosciences). Reactions were cycled for 2 min at 95 °C followed by 30 s at 95 °C, 30 s at 62 °C, and 30 s at 68 °C for 25 cycles followed by 1 min at 56 °C and 20 min at 68 °C. Following capillary electrophoresis and quantification using Genescan software (Applied Biosystems), the ratio of the area under the test peak to the reference peak was taken as an estimate of relative copy number. Each reaction was performed five times with the average of the five ratios taken to represent the relative copy number.

### Droplet Digital PCR

We used TaqMan chemistry to distinguish emulsion droplets positive for the *DEFB2L* amplicon and droplets positive for the *PAX9* gene. Primers specific for *DEFB2L* gene were designed using rhesus macaque rheMac2 reference sequence: Forward primer 5′-TGATATAAGGAATCCTGTTACCTGC-3′, reverse primer 5′-ATGGCTTTTTGCAGCATTTT-3′, and probe 5′-[FAM]GCCATATGTCATCCAGGCTT-[MGB]-3′. Primer sequences and fluorescent probe sequence for the macaque reference diploid gene *PAX9* on chromosome 7 were taken from published data (Lee et al. 2008). For the droplet digital PCR (ddPCR), each reaction was prepared in a final volume of 22 µl, containing 900 nM of each primer, 250 nM of each probe, 11 µl of 2 × ddPCR Supermix (Biorad), and 10 ng of DNA and emulsion prepared according to the manufacturer’s instructions. Forty microliters of the emulsion obtained per each sample was amplified to end point using the following cycling conditions: 95 °C for 10 min followed by 40 cycles of 30 s at 94 °C, 60 s at 56 °C, and finally 10 min at 98 °C. Reads with more than 10,000 droplets generated were considered acceptable. Each reaction was performed four times with the average of the four ratios taken to represent the relative copy number.

### Identification and Characterization of LINE and SINE Insertions

We identified repeat elements from rhesus macaque bacterial artificial chromosome (BAC) sequence data initially using Repeatmasker Web Server, categorizing the sequence as human. The repeat sequences distinguishing the paralogous copies of the defensin duplication were assigned to macaque repeat families by alignment using ClustalW2 (Larkin et al. 2007) and manual comparison of the sequence with published consensus sequences (Liu et al. 2009).

### Copy Number Calling

Using the information on CNV boundaries generated by BreakPtr, we took all aCGH probe values between those boundaries and, for each of the 16 rhesus macaque samples, generated a value for the first principal component of the intensity data. These values were plotted against ddPCR and PRT values for the same 16 samples, and *r*^2^ values calculated (supplementary fig. S4*a*, Supplementary Material online). All the methods were correlated, strongly suggesting that they were all measuring copy number, with the highest correlation coefficient between PRT and the first principal component of the aCGH data. The first principal component of the ddPCR, PRT, and aCGH values was then calculated to generate a single figure, reflecting copy number, for each sample. In the absence of known “gold standard” reference samples, integer copy number calls were manually estimated by comparing this value to ddPCR (supplementary fig. S4*b*, Supplementary Material online). ddPCR, although less precise than PRT based on repeat measurements, appears to be more accurate and results seem to cluster about integer copy numbers, at least for copy numbers 3, 4, and 5. This increased accuracy may be because it is a digital PCR approach, which calculates absolute number of molecules of a given sequence in DNA sample rather than relying on relative amplification (Hindson et al. 2011; Pinheiro et al. 2011).

### BAC Identification and Analysis

Two probes specific for the *DEFB2L* region spanning chr8:8068951–8069850 and chr8:8072076–8073004 were generated by PCR from rhesus macaque genomic DNA and used to probe Segment 1 CHORI-250 BAC library filters (BACPAC Resources Center). In total, 21 positive clones were identified, of which six were selected for further analysis. BAC DNA was extracted from growing *Escherichia coli* cultures using cesium chloride ultracentrifugation, and end sequences generated using T7 and SP6 sequencing primers. Fluorescent in situ hybridization (FISH) with BAC 47B11 shows the main site on the distal end of the short arm of chromosome 8 (fig. 2*C* and *D*) with secondary and some dispersed signal due to repetitive elements contained within the BAC, whereas the probe for *DEFB2L* showed a signal only on chromosome 8. At interphase, one or two variably condensed domains are visible with chromosome 8 paint with the *DEFB2L* probe at the outside of the domain orientated to the interior of the nucleus (fig. 2*F* and *G*).

### Fluorescent In Situ Hybridization

Somatic chromosome preparations were obtained from rhesus macaque lymphoblastoid cell lines 2BX, r00068, and 2BZ (gift from Dr Gaby Doxiadis) after colcemid arrest, hypotonic treatment with 0.075 M KCl and fixation in 100% methanol:glacial acetic acid (3:1) followed standard procedures (Schwarzacher and Heslop-Harrison 2000).

Probes used were human paint chromosome 8 red (PH8RD; Chrombios GmbH, Raubling, Germany), BAC 201P10, BAC 47B11, and four PCR products that together span 4.9 kb of the *DEFB2L* duplication (without encompassing repeated elements) amplified using BAC 47B11 as template. Probes were labeled with biotin dUTP using BioPrime DNA Labelling System (Invitrogen, 18094-011) or with digoxigenin-11dUTP (alkali stable; Roche Applied Sciences) using BioPrime CGH Genomic Labelling System (Invitrogen, 18095-011) following the manufacturer’s instructions.

FISH was performed as previously described (Schwarzacher and Heslop-Harrison 2000). Briefly, slides were pretreated with RNAse (100 μg/ml) and fixed in 4% saline-buffered paraformaldehyde solution before dehydration in an ethanol series. The hybridization mixture contained 40–100 ng probe DNA, optionally 1 μg human Cot-1 DNA (Invitrogen); 25 ng salmon sperm DNA, 40% (v/v) formamide, 1 × SSC, 1.25 mM EDTA and 10% (w/v) SDS and 20 μl was applied to the centre of each slide and covered with a small plastic coverslip. Following denaturation, hybridization overnight at 37 °C, and stringent washing, hybridization sites were detected with Fluorescein (FITC)-conjugated antidigoxigenin (2 µg/ml; Roche Applied Sciences) and Alexa Fluor 546 streptavidin (1 µg/ml; Life Technologies) before staining with DAPI (4′,6-diamidino-2-phenylindole) and mounting in antifade solution (R1320, Agar Scientific). Preparations were analyzed with a Nikon ECLIPSE N80i fluorescent microscope equipped with a DS-QiMc monochromatic camera. Each metaphase or interphase was captured in three different filter sets and then overlaid and analyzed using Adobe Photoshop CS3 or NIS-Elements BR3.1 software (Nikon).

## Results

### The Human β-Defensin CNV Region Is 322 kb in Size and Includes Several Other Genes

At the start of this study, it was known that the β-defensin CNV varied as a continuous block in humans, although the size of the contiguous block was not clear. Previous estimates of the size of the contiguous block were based on concordance of copy number assay results spanning 85 kb from *DEFB107* to *DEFB4* (Groth et al. 2008), although a recent study extended this to 157 kb (Taudien et al. 2014) and pulsed-field gel electrophoresis data suggest that the repeat unit is at least 250 kb (Hollox et al. 2003). To address this, we designed a tiling oligonucleotide array with 190,240 probes covering the region between chr8:6185000 and chr8:84681910 (hg18, supplementary fig. S1, Supplementary Material online) and performed aCGH on a series of 68 DNA samples with known copy number (supplementary table S1, Supplementary Material online). Initial inspection confirmed the CNV of the β-defensin region, together with the more complex CNV of the REPD region flanking the β-defensin region (supplementary fig. S1, Supplementary Material online). Consistent with previous results, we saw no convincing copy number heterogeneity across the region, with the exception of a loss of signal observed in the intron of *DEFB107* in 12 of the 68 individuals. This was confirmed by PCR to represent an absence of the HERVK-115 element, a known polymorphic retrotransposon position in humans (Turner et al. 2001)*.* This element has a frameshift substitution which results in disruption of the *pro* and *pol* coding regions, although it encodes a potentially full-length *env* protein (Turner et al. 2001).

**Fig. 1.—:**
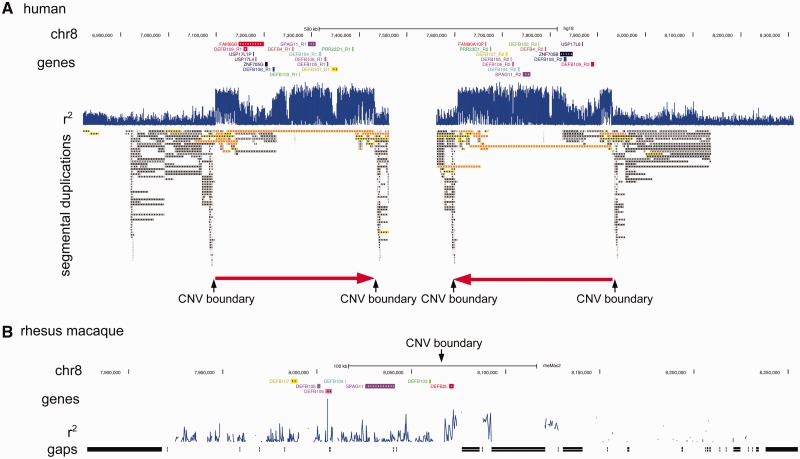
Analysis of CNV of β-defensin regions in humans and rhesus macaque. (*A*) Human. The correlation of each individual arrayCGH probe with the copy number of 68 samples estimated by PRT is shown as the track r^2^cn. Also shown are β-defensin genes mapping to the region, segmental duplications as defined by Bailey et al. (2001), and genomic position. The red arrows indicate the copy number variable repeat (322 kb). (*B*) Rhesus macaque. The correlation of each individual arrayCGH probe with the copy number of 16 samples estimated by PRT and ddPCR is shown as the track r^2^cn. Also shown are Ref-Seq genes in the region, including the *DEFB4* ortholog *DEFB2L*. Location of the putative ortholog of the other human β-defensins mapping to this region is shown in supplementary table S2, Supplementary Material online.

Because the human β-defensin CNV block is embedded within repeat-rich regions REPP and REPD, methods that identify CNV regions based on the transition from normal diploid copy number to variable copy number are unlikely to be effective. Instead, in order to test the extent of the human β-defensin CNV contiguous block, we calculated the squared correlation coefficient (*r*^2^) pairwise between the log_2_ratio for each aCGH probe and the β-defensin copy number determined by triplex PRT, across all 68 samples. PRT is a form of quantitative PCR where test and reference loci are amplified using the same pair of primers designed on a dispersed, divergent, repeat (Aldhous et al. 2010). The rationale behind this approach is that intensity values from aCGH probes that are measuring the same CNV as the PRT will, on average, be strongly correlated with copy number measured by PRT, across a large number of samples. Conversely, those intensity values from aCGH probes outside the CNV region measured by PRT will not be strongly correlated with copy number measured by PRT. Importantly, this last tenet holds whether the aCGH probes map to a diploid non-CNV region or a more complex CNV unrelated to the CNV measured by the PRT. These *r*^2^ values were plotted against the two assembled β-defensin repeats present in the human reference genome and showed a contiguous region of 322 kb where the log_2_ratio of the aCGH probes is correlated with the β-defensin copy number (fig. 1*a*). This region includes the defensin genes *DEFB4*, *DEFB103*, *DEFB104*, *DEFB105*, *DEFB106*, and *DEFB107* as expected, as well the sperm-associated glycoprotein *SPAG11* and the proline rich 23 domain containing one gene (*PRR23D1*). *SPAG11* is related to the β-defensin genes, and is both antimicrobial and necessary for the initiation of sperm maturation (Horsten et al. 2004; Zhou et al. 2004). *PRR23D1* is transcribed and predicted to encode a protein, as yet of unknown function. Other human *PRR23* family members (*PRR23A*, *PRR23B,* and *PRR23C*) are testis-specific genes, according to the RNA sequencing (RNA-Seq) data provided by Illumina BodyMap 2, strongly suggesting that this family has a role in the male reproductive system.

Within the 322 kb contiguous region is a small section which shows a lower level of correlation, due to it being comprised of a low copy repeat that also maps to chromosome 4p16.1, 11q13.4, and 12p13.31. This small section contains *DEFB109, FAM90A10* (Bosch et al. 2007), and *FAM66B* gene families, as well as a *ZNF705* gene, members of the KRAB-associated zinc-finger family of transcription factors (Huntley et al. 2006), and members of the *USP17L* family of deubiquitinating enzymes (Burrows et al. 2005). This is consistent with it being a core duplicon at the center of other distinct CNV regions in the genome (Marques-Bonet and Eichler 2009). According to RNA-Seq data, *DEFB109* is strongly expressed in not only the testis but also the colon and adrenal gland, *FAM66B* in a noncoding RNA fairly ubiquitously expressed but particularly in brain, prostate and breast, and the *ZNF705* genes are expressed in the testis. *FAM90A10* is annotated as a pseudogene, but variants predicted to be active exist, and have been shown to be ubiquitously expressed (Bosch et al. 2007), *USP17L4* is expressed predominantly in the brain and *USP17L8* in the brain and testis. Together with the fact that the *DEFB104*, *DEFB105*, *DEFB106*, *DEFB107*,** and *DEFB108* show strong expression in the testis (García et al. 2001; Yamaguchi et al. 2002; Semple et al. 2003) and a mouse knockout of *DEFB105*, *DEFB106*,** and *DEFB107* shows male infertility (Zhou et al. 2013), our data strongly point to a role of this gene cluster in male reproduction. This raises the question of the role of this CNV in modulating normal male fertility.

### The Rhesus Macaque β-Defensin CNV Consists of a 20-kb Tandemly Repeated Unit Containing Only DEFB2L

We designed an array to investigate the nature and extent of CNV of the β-defensin locus in rhesus macaques. Using the region defined previously as being CNV (Lee et al. 2008; Gokcumen et al. 2011), we designed 875 probes spanning a region of 982 kb on the rheMac2 genome assembly, and performed aCGH on 16 unrelated rhesus macaque samples. It should be noted that this assembly contained a substantial number of gaps, complicating probe design and resulting in areas with no coverage, but with a coverage density of approximately 1.5 probes per kb in assembled regions. We also identified and mapped the β-defensin genes orthologous to those in the human β-defensin repeat and confirmed conserved order and orientation of the genes from *DEFB107* to *DEFB2L* (the ortholog of human *DEFB4*) in the macaque genome (supplementary table S2, Supplementary Material online). We used a hidden Markov Model approach, implemented in the software BreakPtr (Korbel et al. 2007), to search for a potential CNV boundary between the two genes in our aCGH data, and identified a CNV boundary between chr8:8069931 and chr8:8071942 reflecting normal copy number distally but loss of DNA proximally.

**Fig. 2.—:**
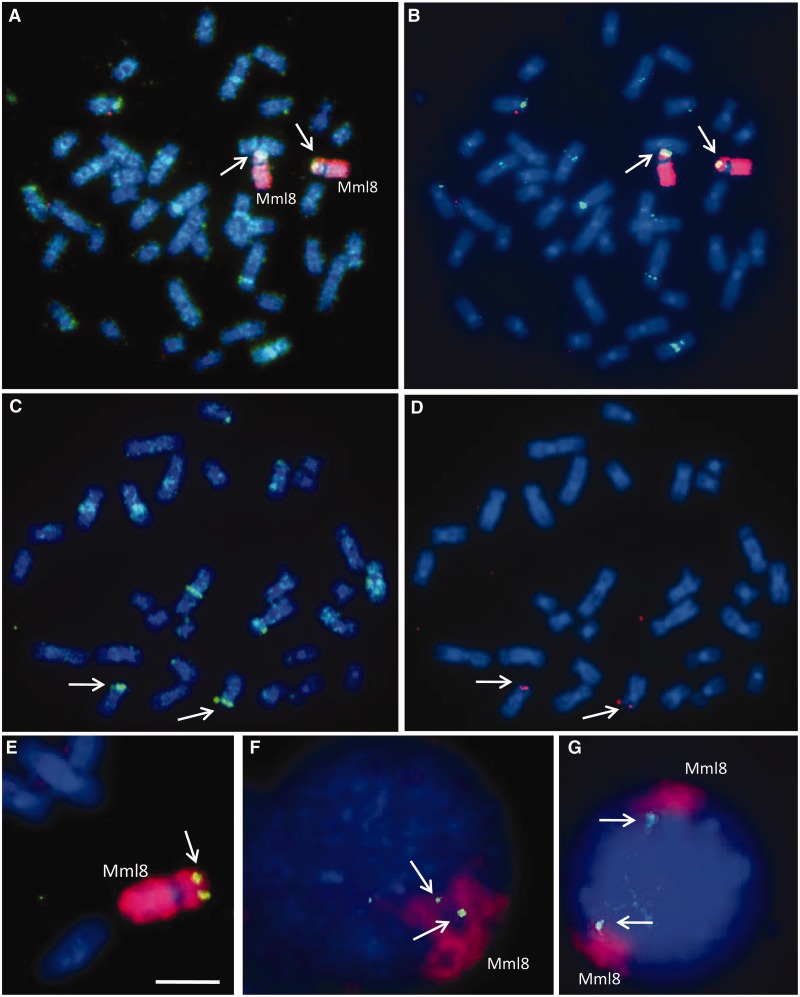
Localization of *DEFB2L* in rhesus macaque by FISH analysis. Double target FISH of BACs 201P10 and 47B11 together with chromosome 8 paint from human and the gene of interest probe *DEFB2L* to somatic rhesus macaque metaphase and interphase chromosomes stained blue with DAPI. (For probe description, see Materials and Methods.) (*A*, *B*) BAC 201P10 shows strong signal (digoxigenin-FITC, green, arrow) on the short arm of macaque chromosome 8 (Mml8) that is characterized by the human chromosome 8 paint (red). Many chromosomes also show dispersed signal (*A*) that is consistent with the BAC containing retroelements and other repeats that are not observed under stringent image analysis (*B*). The strong sites on Mml8 and some secondary sites on other chromosomes are clearly visible. (*C*, *D*) BAC 47B11 (digoxigenin-FITC, green, *C*) shows few dispersed sites and the main locus on the distal end of the short arm of chromosome 8 (arrows); a few stronger secondary sites are also visible. *DEFB2L* shows pairs of dots (Biotin Alexa 546, red; arrows in *D*) in the same region of chromosome Mml8 as the BAC 47B11 probe. (*E*) Metaphase chromosome Mml8 identified by red fluorescence of the human chromosome 8 paint (red) shows a single pair of dots from *DEFB2L* (digoxigenin-FITC yellow green, arrow) at the distal end of the short arm. (*F*, *G*) Interphase nuclei probed with human chromosome 8 paint (red) and *DEFB2L* (digoxigenin-FITC green). One (*F*) or two (*G*) domains of various compactions are visible with the defensin gene at the edge of the domains orientated toward the interior of the nucleus. Bar 10 µm in (*A*)–(*G*) and 5 µm in (*E*).

**Fig. 3.—:**
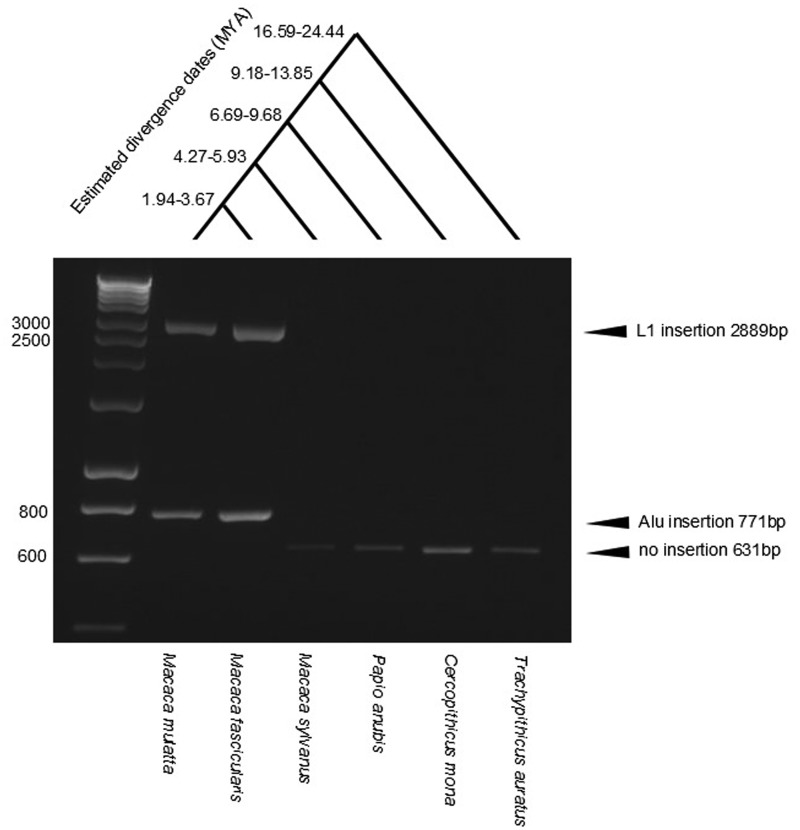
Estimation of the most recent possible date of *DEFB2L* duplication by analysis of retroelements insertions. PCR analysis of the presence or absence of an *Alu* insertion and an L1 insertion in the 20-kb β-defensin repeat. Using primers designed on rhesus macaque sequence, amplicons from Old World monkeys with increasing divergence dates from rhesus macaque are shown. Estimated divergence dates are from Perelman et al. (2011). The numbers on the left indicate sizes of DNA molecular weight markers, in base pairs.

In parallel, we isolated BAC clones from a rhesus macaque genomic library using two sequences as probes (chr8:8068951–8069850; chr8:8072076–8073004) both in vitro by probing arrayed-BAC filters and in silico by database searching. We characterized six BACs containing the *DEFB2L* region using a combination of BAC end sequencing, PCR analysis, and FISH (supplementary table S3, Supplementary Material online), confirming that *DEFB107*–*DEFB2L* were arranged as predicted in the rheMac2 genome assembly (supplementary table S2, Supplementary Material online), and that *DEFB2L* mapped uniquely to distal 8p (fig. 2). FISH also confirmed that the BACs mapping to this region contain many dispersed repeats, and that at interphase *DEFB2L* is at the edge of the chromosome 8 domain. The full Sanger sequence of two further BACs from the same library (CHORI-250) was available (BAC 243E20 accession AC191454.4, BAC 65I2 accession AC193549.4) and analysis of these sequences showed two copies of the *DEFB2L* gene on a 20-kb tandem duplication (supplementary fig. S2, Supplementary Material online), with a distal boundary of the duplication consistent with the CNV boundary identified from aCGH data. The proximal boundary of the duplication, identified in 243E20, is not assembled on chromosome 8 in the rheMac2 assembly, illustrating the limitations of using a whole-genome shotgun assembly for array CGH design and the importance of BAC sequencing in assembling complex genomes. The two full-length copies on 243E20 share 91.3% identity at the nucleotide level, and we can estimate that the duplication event occurred around a similar time to divergence of the macaque lineage from baboon (Elango et al. 2009), about 10 Ma (Raaum et al. 2005). However, directly dating the origin of the duplication is hampered by the lack of convincing orthologous sequences for the entire 20-kb repeat from other Old World monkeys, and the sequence homogenizing influence of gene conversion. Fortuitously, in BAC 243E20, the two full-length paralogs are distinguished by an AluYRa1 SINE insertion on the proximal copy and an L1PA5 LINE insertion on the distal copy. The AluYRa1 subfamily has been estimated to be approximately 9.5 Ma old (Han et al. 2007; Liu et al. 2009), putting the earliest origin of the duplication at that point. By designing primers matching the sequence flanking these insertions, we can identify the presence of the duplication using PCR (fig. 3). Analysis of other Old World monkeys with known divergence times strongly suggests that two paralogous copies distinguished by the LINE and SINE insertions arose after the divergence of the lineage leading to *Macaca sylvanus* (∼4 Ma), but before the divergence of the lineage leading to *M. fascicularis* (∼3 Ma). This suggests that the duplication could be as recent as 3 Ma (fig. 3). In *M. mulatta*, paralogs without the integration of the LINE or SINE are seen in some individuals, confirming that the integrations occurred after the initial increase in copy number.

Initial studies using real-time quantitative PCR approaches had suggested that, in rhesus macaques, *DEFB103* showed a constant diploid copy number of 2 per diploid genome, whereas *DEFB2L* showed CNV (supplementary fig. S3, Supplementary Material online). With a working hypothesis that the 20-kb tandem duplication reflected the unit of CNV, we designed two PCR-based assays to measure the copy number of this duplication. The first assay is a PRT (Armour et al. 2007; Veal et al. 2013). The second assay, based on ddPCR, uses an emulsion PCR approach to create many thousands of microvolume PCR reactions, with droplets counted at endpoint for presence or absence of the test and reference PCR products. Using a combination of the two PCR assays, together with the first principal component of the aCGH intensity data spanning the *DEFB2L* duplication, we estimated the likely diploid copy number for the *DEFB2L* gene in the rhesus macaque samples, which ranges between three and six copies per diploid genome (supplementary fig. S4, Supplementary Material online). The multiallelic nature of the rhesus macaque *DEFB2L* CNV strongly suggests that duplication of *DEFB2L* has been a recurrent event within the macaque lineage.

Using this information on the copy number of the 16 samples, we plotted the square of the correlation coefficient (*r*^2^) pairwise between the log_2_ratio for each aCGH probe and the β-defensin copy number across the 16 macaque samples. Despite a higher background noise, partly due to fewer probes and smaller sample size, a boundary corresponding to the distal end of the *DEFB2L* duplication can be seen, in agreement with the BreakPtr analysis (fig. 1*b*). This analysis confirms that the CNV region does not include the other β-defensin genes, only *DEFB2L*, and is very likely to correspond to the size of the duplication (20 kb).

### Different Endogenous Retroviral Long Terminal Repeat Sequences Were Involved in the Initial Mutational Event Creating the CNV in Humans and Rhesus Macaques

**Fig. 4.—:**
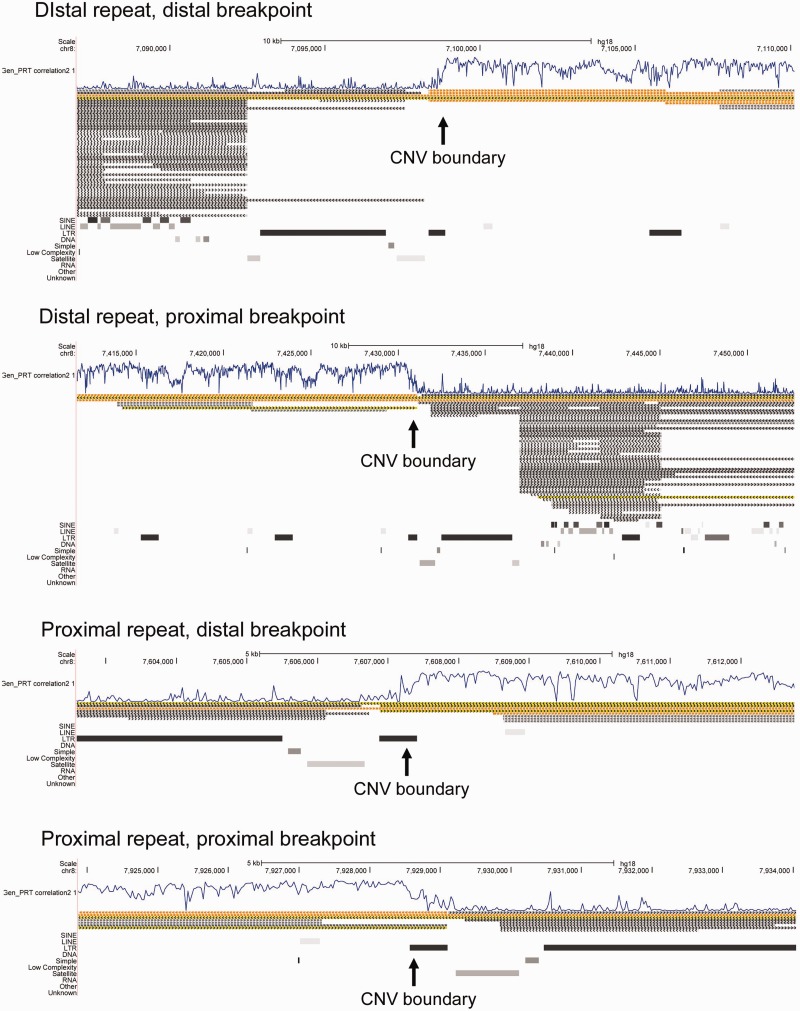
Analysis of human CNV boundary regions. The four panels show the hg18 reference genome (UCSC genome browser) at the distal and proximal boundaries of the distal and proximal assembled β-defensin repeat regions (see fig. 1). In all four cases, the boundary of the β-defensin CNV, as measured by the correlation with copy number measured by PRT (track “Gen_PRT correlation 2”), is at the LTR65 element.

Analyzing the β-defensin CNV boundary in both macaques and humans is of interest because it might give insights into the mutational history of the locus. Using the high-resolution aCGH data, we inspected the β-defensin CNV boundaries, defined by an abrupt change in the *r*^2^ value of the log_2_ratio for each aCGH probe and the β-defensin copy number determined by the PRT. In this study, the abrupt changes in the *r*^2^ value were identifiable by visual inspection (figs. 1*a* and 4) but in future studies such changes could be identified automatically using hidden-Markov model type approaches.

In humans, analysis of both assembled regions shows that at the boundary between the β-defensin CNV and more complex flanking regions is a section of a long terminal repeat (LTR) (LTR5A) of the ape-specific endogenous retrovirus family ERVK (Repeatmasker database [Smit et al. 2010], http://www.repeatmasker.org, last accessed October 29, 2014), organized antiparallel to each side of the CNV region (fig. 4). The antiparallel arrangement of the LTR reflects the antiparallel arrangement of the complex segmental duplication-rich region flanking the CNV boundaries, a region which contains olfactory receptor genes. This arrangement suggests that this region is closely tied to the polymorphic recurrent inversion of 8p23.1, which is known to involve the REPP and REPD regions (Giglio et al. 2001; Salm et al. 2012).

In rhesus macaques, the ends of the tandem repeat can be defined more precisely since, at least for the distal boundary, the transition is to single copy sequence (rather than more complex CNV regions as observed in human). By analyzing BAC 243E20 and 65I2 sequences, and aCGH data (supplementary fig. S5, Supplementary Material online), the boundary can be defined as part of a tandemly organized LTR (LTR65) of the endogenous retrovirus family ERV1, found in all eutherian mammals.

### Paralogous and Allelic Variants of *DEFB2L* Are Expressed in the Gut and Have Undergone Natural Selection

*DEFB2L* is expressed in the rhesus macaque gastrointestinal tract, and previous sequencing of cDNA derived from gastric biopsies has revealed new transcripts with nonsynonymous substitutions (DEFB2L1–L5 [Hornsby et al. 2008], table 1). Here we report two new variants identified from further transcript sequencing (DEFB2L6 and DEFB2L8), defined by variation at nonsynonymous sites. The advantage of sequencing transcripts is that it shows that the variants are actively transcribed and are not likely to be pseudogenes. However, such analysis does not establish whether the variation observed is due to sequence differences between closely related paralogs (paralogous sequence variant), between alleles (single nucleotide polymorphism), or between paralogs and alleles (multisite variant). Because each individual BAC is derived from one of the two chromosome 8 homologs, identification of variants between *DEFB2L* copies in a BAC shows that these variants occur in *cis*, that is, between true paralogs. In silico analysis of the two BAC DNA sequences from the database (BAC 243E20 and BAC 65I2) confirmed the existence of two variants (DEFB2L7 and DEFB2L6; table 1). In addition, we could identify DEFB2L6 and DEFB2L7 (BAC 243E20), together with DEFB2L1 and DEFB2L7 (BAC 65I2) as true paralogs.

**Table 1:** Rhesus Macaque DEFB2L Variants

Variant	Unambiguously Observed in	Number of Amino Acid Changes	Amino Acid Changed	Previously Reported?
DEFB2L7	Transcript, 65I2^a^, 243E20^a^, 246K23, 47B11, 201P10	0	0	Reference genome rheMac2
DEFB2L1	Transcript, 65I2^a^	4	G45R, V52I, P56S, L57A	Hornsby et al. (2008)
DEFB2L6	Transcript, 243E20^a^	3	G45R, P56S, L57A	Novel
DEFB2L2	Transcript only	1	L32I	Hornsby et al. (2008)
DEFB2L3	Transcript only	4	F22S, G45R, P56S, L57A	Hornsby et al. (2008)
DEFB2L4	Transcript only	4	F12L, G45R, P56S, L57A	Hornsby et al. (2008)
DEFB2L5	Transcript only	4	G45R, P56S, L57A, K63N	Hornsby et al. (2008)
DEFB2L8	Transcript only	6	F12L, R26G, S34G, G45R, P56S, L57A	Novel

^a^BACs informative for the discrimination of paralogous and allelic DEFB2L variants.

**Fig. 5.—:**
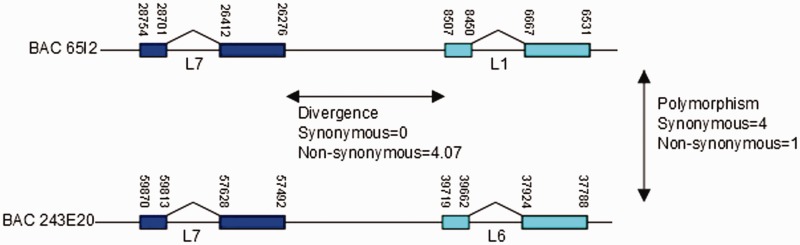
Using the McDonald Kreitman test to detect selection between rhesus macaque *DEFB2L* paralogs. The two fully sequenced BACs, each with two tandem 20-kb *DEFB2L* repeats, are shown. BAC 65I2 has *DEFB2L1* and *DEFB2L7* in tandem, whereas BAC 243E20 has *DEFB2L6* and *DEFB2L7* in tandem. The number of nonsynonymous and synonymous nucleotide changes between paralogs (divergence) and between alleles (diversity) is shown next to the double-headed arrows. Positions of exon-start and -end bases, numbered according to the BAC sequence position, are also shown.

The identification of true paralogs from BAC sequences allows a McDonald–Kreitman test to be constructed comparing the diversity between allelic copies with distance between paralogous copies (fig. 5). Analysis of the four BAC *DEFB2L* coding sequences, with a Jukes–Cantor correction, suggests that positive selection has acted on the two paralogs following duplication (*P* = 0.015), further supporting an adaptive role for amino acid substitutions differing between DEFB2L variants.

## Discussion

Previous work has identified the β-defensin region on chromosome 8 as a CNV hotspot in primates. However, because of uncertainties in the assembly or limitations of aCGH resolution, these studies could not distinguish whether the recurrent CNV is mediated by a shared genomic structure in the hotspot region, characterized by a similar pattern of segmental duplications, or by truly independent events derived from a distinct genomic structure. Our work clearly shows an independent origin of β-defensin CNV in humans and macaques, with the macaque CNV spanning *DEFB2L* (the ortholog of human *DEFB4*), but not the orthologs of other β-defensin genes that show CNV in humans. Moreover, LTRs of different endogenous retroviruses have mediated the initial CNV-forming event, and the allelic extent of CNV in macaques is similar to humans (Hollox et al. 2003; Hardwick et al. 2011) and chimpanzees (Hardwick et al. 2011), which range from three to six copies per diploid genome. Taken together, this provides strong evidence of convergent evolution of *DEFB4* CNV as a molecular phenotype, and argues for selection for variability per se. Yet arguments for the selection of variation are controversial, because it is difficult to envisage how the trait of variability can confer an immediate reproductive advantage. Nevertheless, it can be reconciled if CNV is regarded as a locus with a higher mutation rate generating novel β-defensin copy number alleles favored by frequency dependent selection, for example. Although the mutation rate of the rhesus macaque CNV is not known, family studies have suggested a mutation rate of the human locus of around 0.7% per generation (Abu Bakar et al. 2009). Alleles of different copy number, while clearly inherited in a Mendelian manner, combine to give a diploid copy number genotype which can vary substantially between parent and child, and between siblings, due to normal segregation of homologous chromosomes. It is tempting to speculate that this may be advantageous in animals that are organized in social kinship groups sharing the same environmental exposure to pathogens.

Because gene conversion between segmental duplications maintains sequence identity, it has recently been suggested that this is an explanation of why CNV hotspots, such as the β-defensin CNV hotspot, appear to persist throughout evolution during the divergence of different species (Fawcett and Innan 2013). This model would also argue against an adaptive role for CNV hotspots, which, in such a model, could be a neutral consequence of sequence homogenization of segmental duplications. Our work argues against this model, at least for the persistence of the β-defensin CNV hotspot since the divergence of macaque and human lineages, because the genomic structure sponsoring the CNV is different in macaques and humans. The β-defensin hotspot might be an exception, where truly recurrent CNV formation across different lineages is favored by selection for new functional roles for β-defensins, or modification of existing roles in fertility, signaling and antimicrobial activity. Further high-resolution mapping of other CNV hotspots across mammals is needed to determine whether Fawcett and Innan’s model holds for most CNV hotspots.

The identification of the CNV boundary in humans has two important consequences. First, identification of the boundary within an LTR as part of a large antiparallel segmentally duplicated region suggests a link to the frequent polymorphic inversion at 8p23.1, where the 5-Mb region between REPP and REPD is inverted. This is because inversions are sponsored by recurrent nonallelic homologous recombination between antiparallel repeats. Analysis of potentially recombinant BAC sequences from this region has previously suggested multiple inversion breakpoints, particularly centered on the olfactory repeat region, but the closest is at least 50 kb away from the β-defensin CNV boundaries (Salm et al. 2012). So although it looks very likely that the entire β-defensin copy number region was repeatedly carried between REPP and REPD by inversion formation (Abu Bakar et al. 2009; Salm et al. 2012), the relationship between the inversion and CNV remains unclear, and is worthy of further study.

The second important consequence of the identification of the CNV boundary in humans is that nondefensin genes within the repeat unit that are confirmed as copy number variable may show alteration of expression levels concomitant with the CNV. Several of these genes are either exclusively expressed or most strongly expressed in the testis. Unfortunately, robustly testing the dependence of copy number with expression levels will be challenging because of limitations of tissue sampling. Despite that challenge, determining the function of these genes, as well as the association of male fertility phenotypes with both copy number and sequence variation, should be a research priority.

We also observed evidence of positive selection on *DEFB2L*-coding sequences after duplication in macaques. This is not observed in humans, where common coding variation has not been found at *DEFB4* (and indeed at most other defensin genes in the repeat unit). Instead, in humans, it seems that some of the sequence variants between copies affect the level and pattern of gene expression in both *DEFB4* (Groth et al. 2008) and *DEFB103* (Hardwick et al. 2011); for *DEFB103,* population diversity data provide modest support for selection on such noncoding elements (Hardwick et al. 2011). We provide evidence that selection in macaques has favored *DEFB2L*-coding sequence divergence after duplication 3–9.5 Ma. The functional basis for this selection is not yet clear; the functions of the DEFB2L proteins are not known, although because of their homology with human hBD2, they are likely to be antimicrobial and immunomodulatory, although perhaps with altered specificities such as has been found in an ortholog of hBD2 from *M. fascicularis* (Antcheva et al. 2004). By comparison with the human hBD2, the mature peptide is encoded by amino acids 28–64. Therefore, all except two of the predicted amino acid changes in the DEFB2L variants are in the mature protein and have the potential to affect function. A preliminary analysis shows that most of these variants lie within a predicted loop connecting two-antiparallel β strands, a region that has been shown by comparative analyses between primates and rodents to be under positive selection (Semple et al. 2005). Also of note is that the L32I change in DEFB2L2 is within a six-amino acid motif that is responsible for hBD2 binding to the chemokine receptor CCR6 (Taylor et al. 2007).

The extensive sequence variation of *DEFB2L* is worthy of further study in wild rhesus macaques and other macaque species, as it may not only provide information on selection but also yield a structure–function analysis of important amino acids in β-defensin 2, which may lead to new therapeutics that could retain, for example, antimicrobial function, but not inflammatory properties.

## Supplementary Material

Supplementary figures S1–S5 and tables S1–S3 are available at *Genome Biology and Evolution* online (http://www.gbe.oxfordjournals.org/).

Supplementary Data
