# Supplementary material for: Evidence of Convergent Evolution in Humans and Macaques Supports an Adaptive Role for Copy Number Variation of the β-Defensin-2 Gene
Source: Genome Biol Evol. 2014 Oct 27;6(11):3025–38. doi: 10.1093/gbe/evu236 (PMC4255768; doi:10.1093/gbe/evu236)
Supplement: Supplementary Data [file supp_6_11_3025__index.html]

Evidence of convergent evolution in humans and macaques supports an adaptive role for copy number variation of the β-defensin-2 gene — Evidence of Convergent Evolution in Humans and Macaques Supports an Adaptive Role for Copy Number Variation of the β-Defensin-2 Gene — Supplementary Data 

# Evidence of Convergent Evolution in Humans and Macaques Supports an Adaptive Role for Copy Number Variation of the β-Defensin-2 Gene

## Supplementary Data

files

**Files in this Data Supplement:**

- Supplementary Data - pdf file
- Supplementary Data - pdf file
- Supplementary Data - docx file
- Supplementary Data - docx file
- Supplementary Data - jpeg file
- Supplementary Data - jpg file
- Supplementary Data - jpg file
