# Supplementary material for: Evidence of Convergent Evolution in Humans and Macaques Supports an Adaptive Role for Copy Number Variation of the β-Defensin-2 Gene
Source: Genome Biol Evol. 2014 Oct 27;6(11):3025–38. doi: 10.1093/gbe/evu236 (PMC4255768; doi:10.1093/gbe/evu236)
Supplement: Supplementary Data [file supp_evu236_supp_figure_1.pdf]

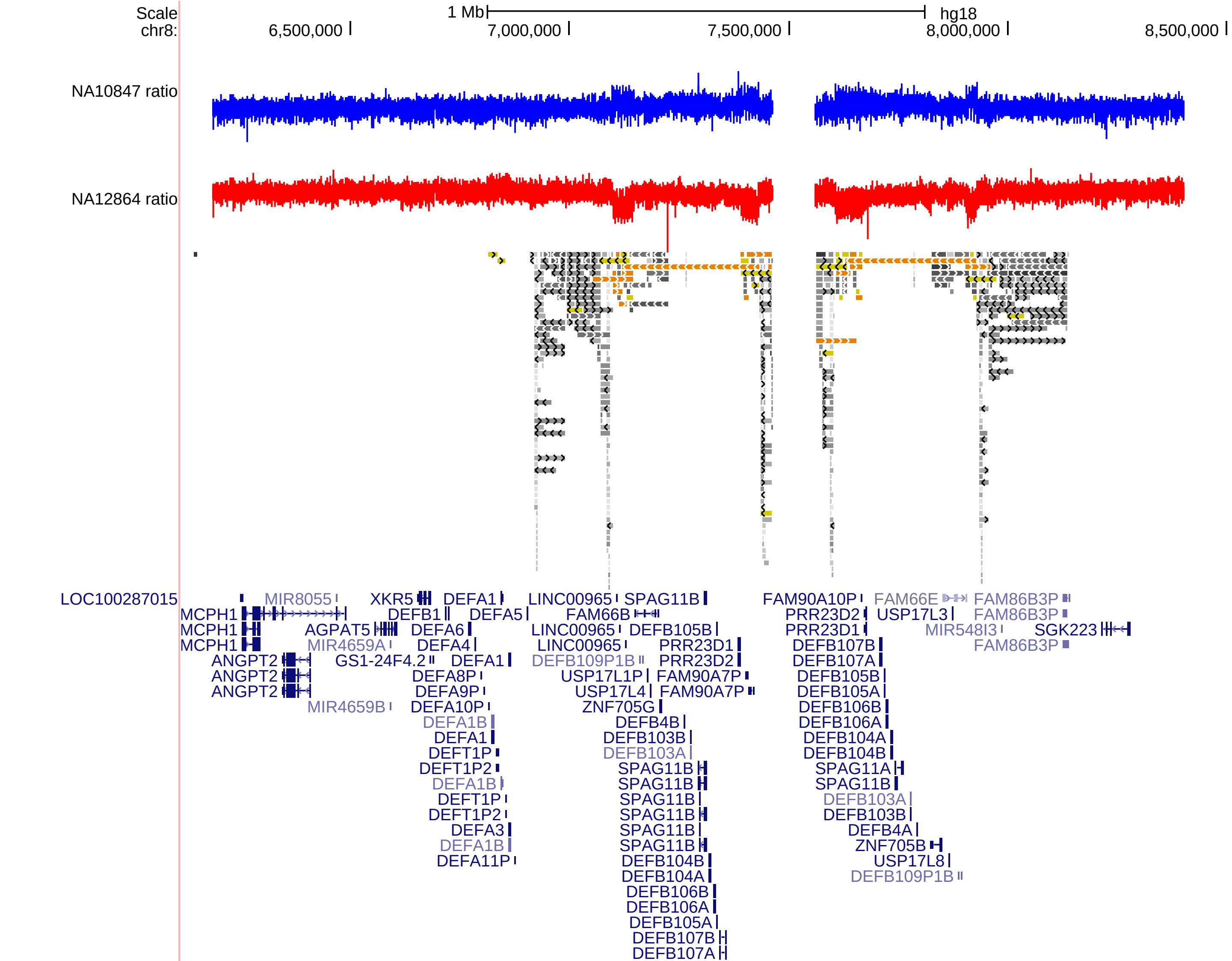
Scale
chr8:
1 Mb
hg18
6,500,000
7,000,000
7,500,000
8,000,000
8,500,000
NA10847 ratio
NA12864 ratio
LOC100287015
MIR8055
XKR5
DEFA1
LINC00965
SPAG11B
FAM90A10P
FAM66E
FAM86B3P
MCPH1
DEFB1
DEFA5
FAM66B
PRR23D2
USP17L3
FAM86B3P
MCPH1
AGPAT5
DEFA6
LINC00965
DEFB105B
PRR23D1
MIR548I3
SGK223
MCPH1
MIR4659A
DEFA4
LINC00965
PRR23D1
DEFB107B
FAM86B3P
ANGPT2
GS1-24F4.2
DEFA1
DEFB109P1B
PRR23D2
DEFB107A
ANGPT2
DEFA8P
USP17L1P
FAM90A7P
DEFB105B
ANGPT2
DEFA9P
USP17L4
FAM90A7P
DEFB105A
MIR4659B
DEFA10P
ZNF705G
DEFB106B
DEFA1B
DEFB4B
DEFB106A
DEFA1
DEFB103B
DEFB104A
DEFT1P
DEFB103A
DEFB104B
DEFT1P2
SPAG11B
SPAG11A
DEFA1B
SPAG11B
SPAG11B
DEFT1P
SPAG11B
DEFB103A
DEFT1P2
SPAG11B
DEFB103B
DEFA3
SPAG11B
DEFB4A
DEFA1B
SPAG11B
ZNF705B
DEFA11P
DEFB104B
USP17L8
DEFB104A
DEFB109P1B
DEFB106B
DEFB106A
DEFB105A
DEFB107B
DEFB107A
