# Supplementary figures and images for: Evidence of Convergent Evolution in Humans and Macaques Supports an Adaptive Role for Copy Number Variation of the β-Defensin-2 Gene
Source: Genome Biol Evol. 2014 Oct 27;6(11):3025–38. doi: 10.1093/gbe/evu236 (PMC4255768; doi:10.1093/gbe/evu236)

A

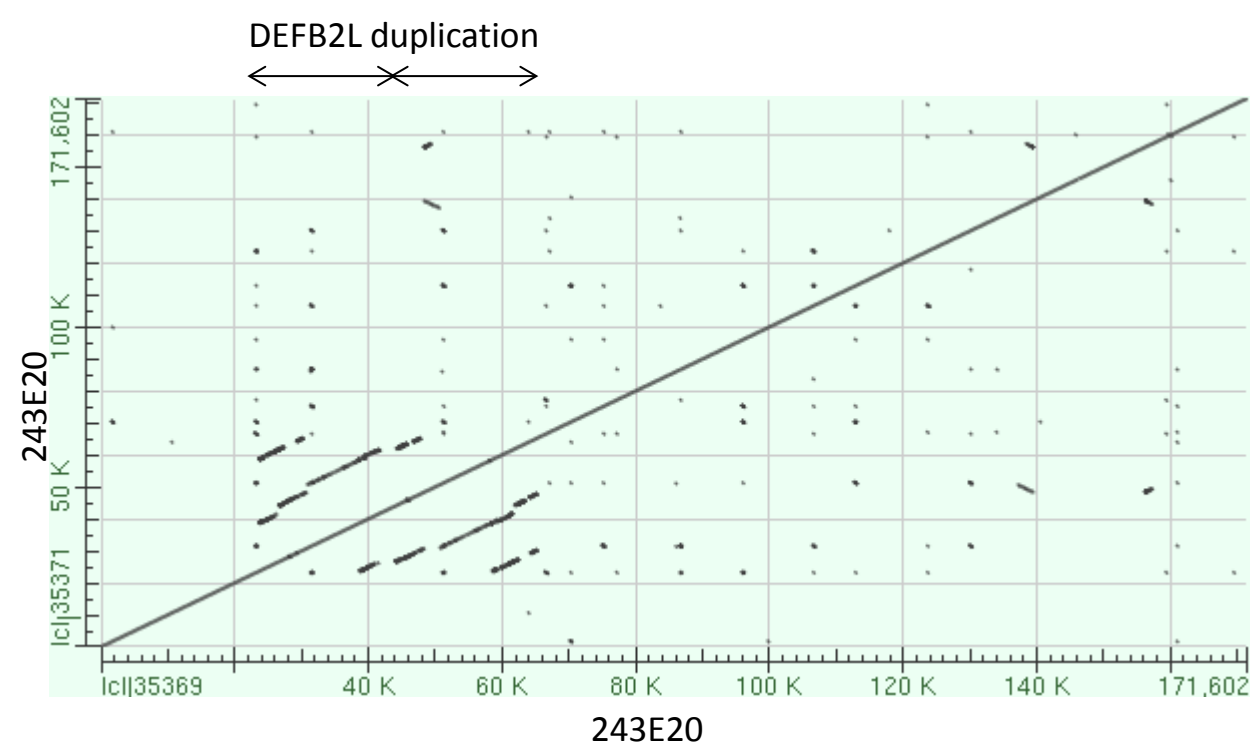

B

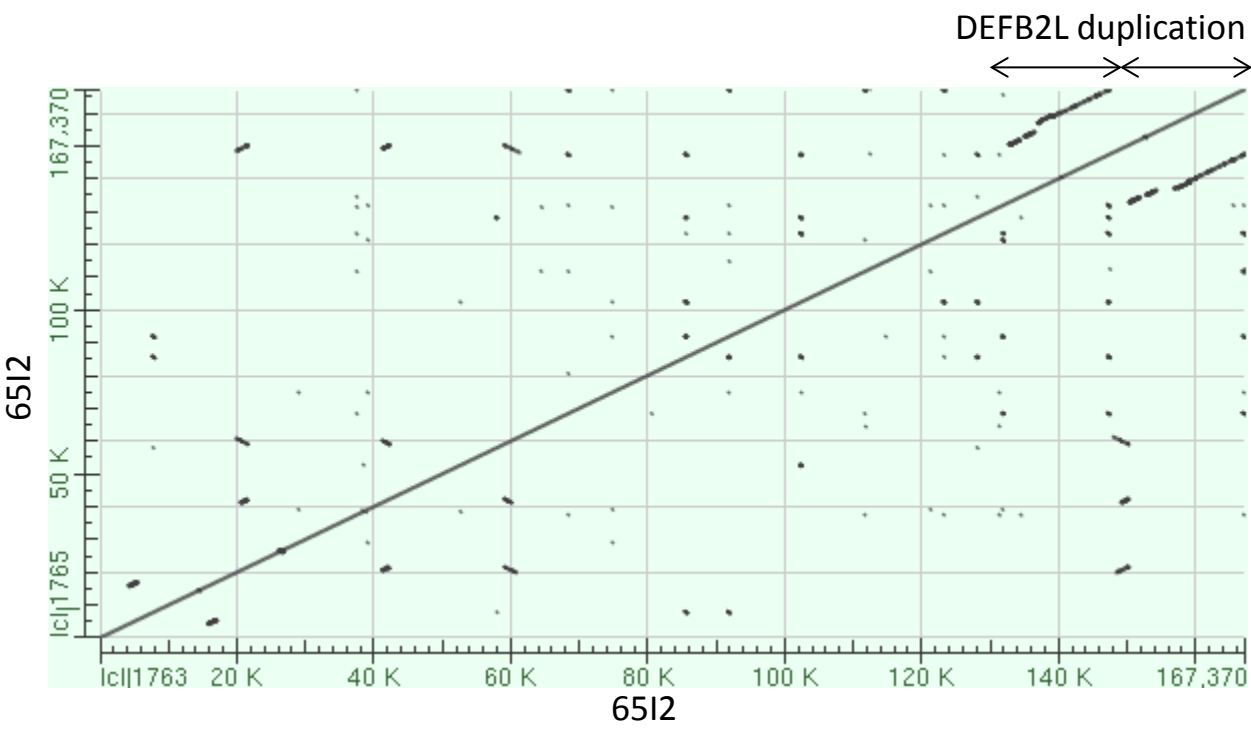

Supplement: Supplementary Data [file supp_evu236_supp_figure_2.pdf]

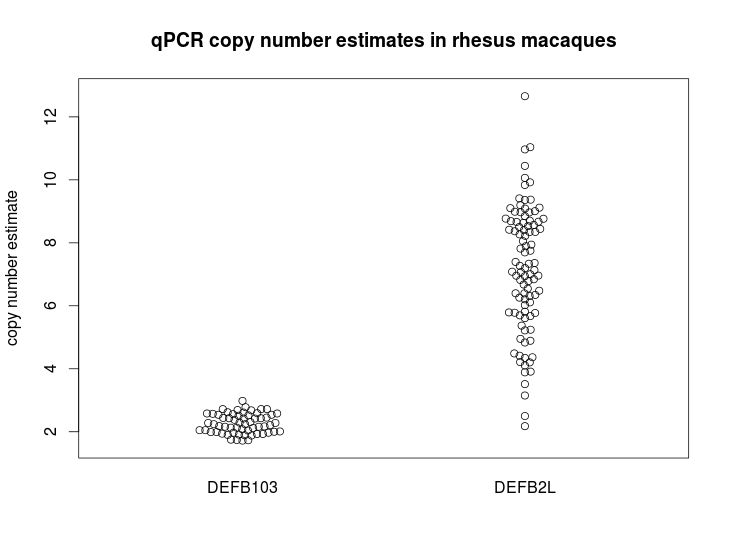

Supplement: Supplementary Data [file supp_evu236_supp_figure_3.jpg]

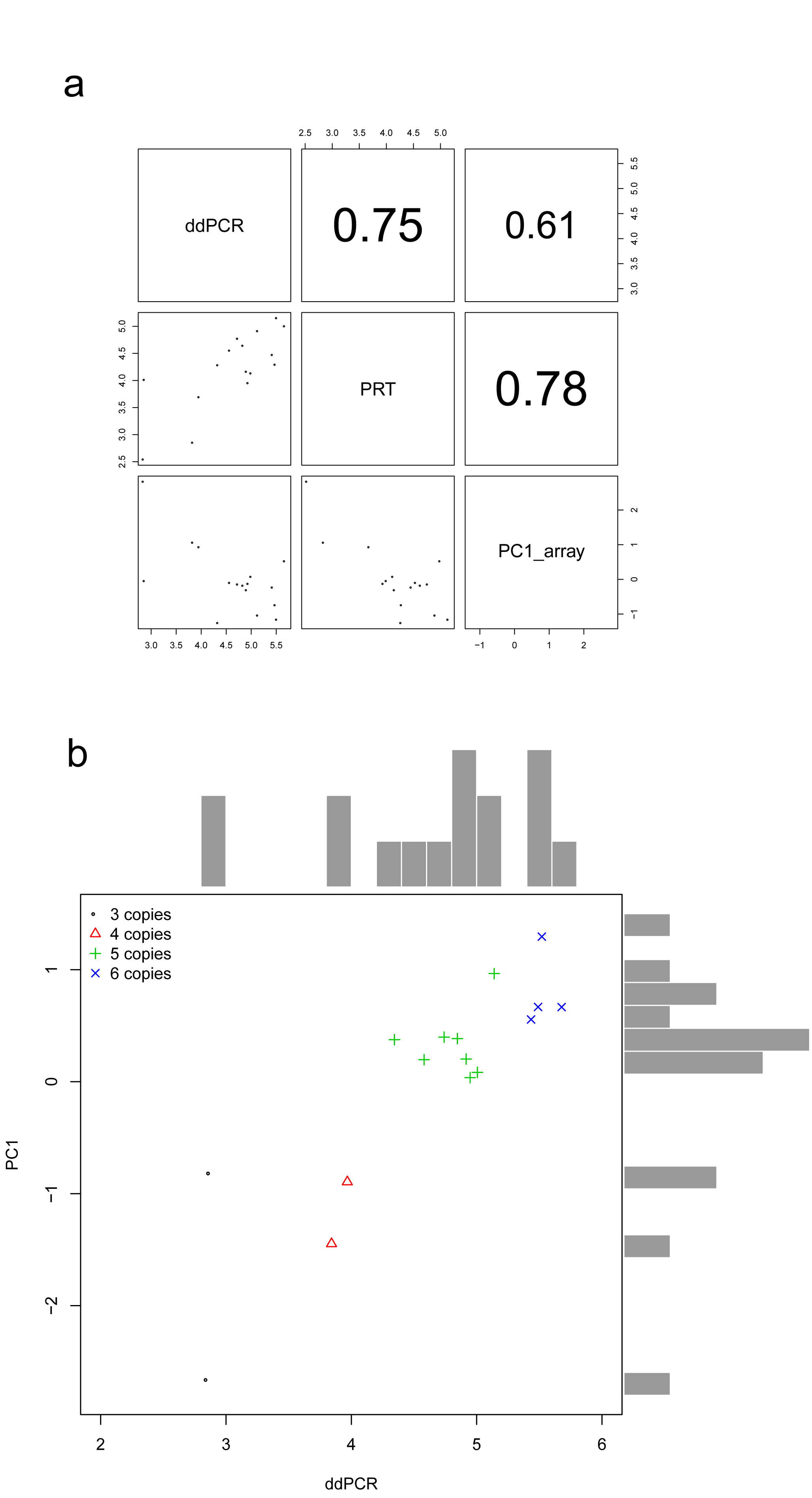

Supplement: Supplementary Data [file supp_evu236_supp_figure_4.jpg]

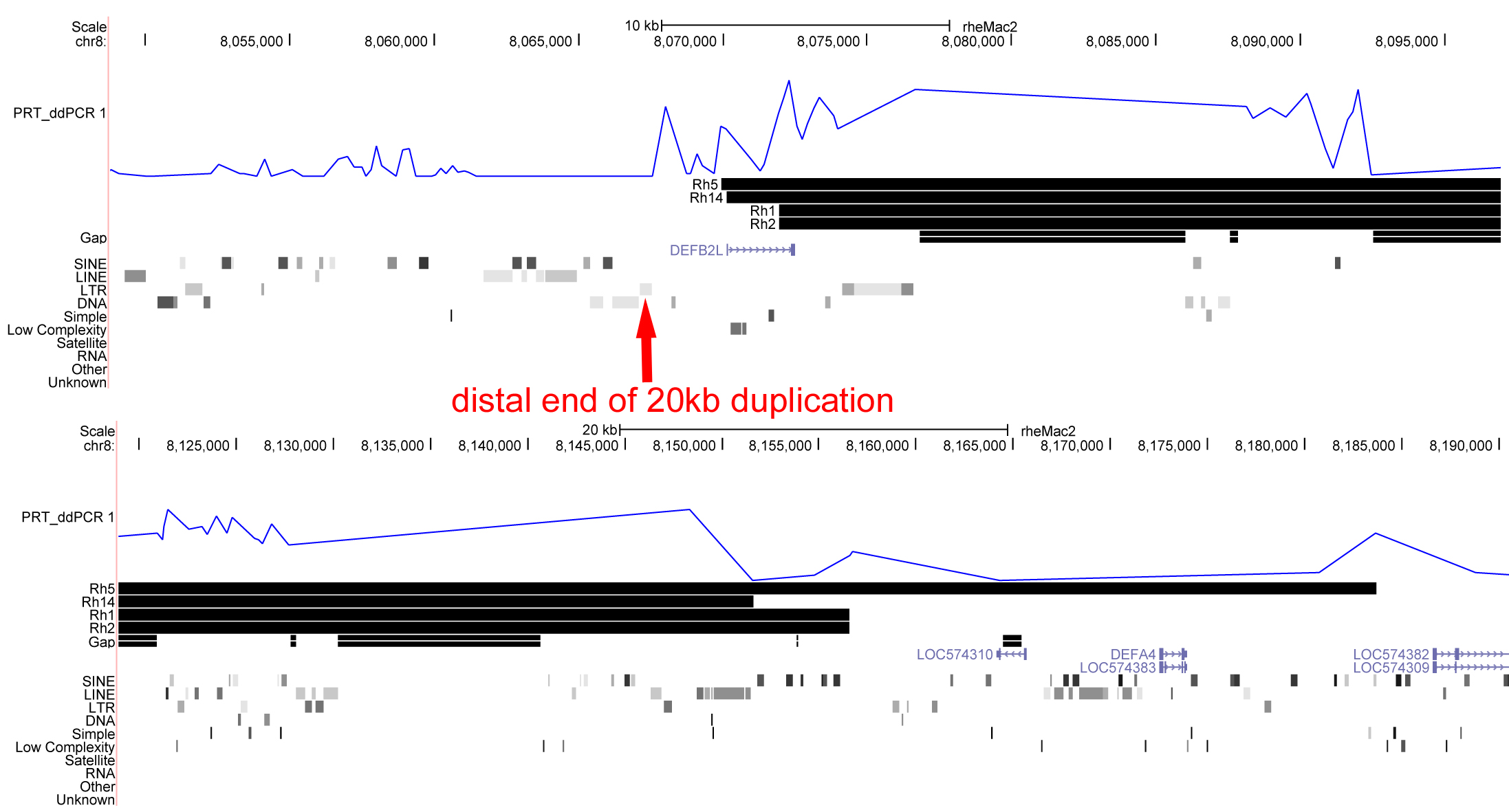

Supplement: Supplementary Data [file supp_evu236_supp_figure_5.jpg]
