# Supplementary material for: Evidence of Convergent Evolution in Humans and Macaques Supports an Adaptive Role for Copy Number Variation of the β-Defensin-2 Gene
Source: Genome Biol Evol. 2014 Oct 27;6(11):3025–38. doi: 10.1093/gbe/evu236 (PMC4255768; doi:10.1093/gbe/evu236)
Supplement: Supplementary Data [file supp_evu236_supplementary_figure_legends.docx]

**Supplementary figure 1 Array CGH analysis of β-defensin region of human chromosome 8**

Normalised aCGH values for two samples, representing the extremes of the observed copy number distribution in the cohort are shown – NA10847 7 copies and NA12864 2 copies. The segmental duplication track is shown, with the β-defensin CNV region highlighted with red arrows. Two copies of the region are assembled in hg18, separated by a gap. Refseq genes are also annotated. Figure generated using the UCSC genome browser ([Kent, et al. 2002](#_ENREF_34)).

**Supplementary figure 2 Dotplot identifying *DEFB2L* duplication in rhesus macaques**

a) BAC 243E20.

b) BAC 65I2. Dotplots generated by blast2seq software.

**Supplementary figure 3 Real-time qPCR estimates in rhesus macaques**

Diploid raw copy number estimates are shown for *DEFB103* (60 individuals) and *DEFB2L* (95 individuals), using β-globin gene region as a reference locus.

**Supplementary figure 4 Calling *DEFB2L* copy numbers in rhesus macaques**

a) Correlation between different methods (digital droplet PCR, paralogue ratio test and aCGH) measuring *DEFB2L* copy number in 16 macaque samples. Numbers reflect pairwise r^2^ values between methods.

b) Scatterplot showing first principal component (PC1) of aCGH, PRT and droplet digital PCR data for each sample, and digital droplet PCR alone. The first principal component is a value on a scale describing the greatest amount of variation across the samples, and can be regarded as a summary value incorporating values from aCGH, PRT and droplet digital PCR data. Assignment of each sample to an integer copy number is shown according to the legend.

**Supplementary figure 5 Analysis of rhesus macaque CNV boundary regions**

Plot of distal (upper panel) and proximal (lower panel) CNV boundaries identified from aCGH data using BreakPtr analysis. The upper blue line indicates the r2 value of each aCGH probe with PRT/ddPCR copy number estimates for the 16 rhesus macaques. The track beneath indicates the samples showing CNV from aCGH signal, as identified by BreakPtr (Rh5, Rh14, Rh1, Rh2). The distal breakpoint is defined with some consistency, and is consistent with the 20kb duplication boundary identified with a red line. Note that the proximal end of the duplication, as defined by BAC243E20, is not assembled at this position in this genome assembly.
