# Supplementary material for: Evidence of Convergent Evolution in Humans and Macaques Supports an Adaptive Role for Copy Number Variation of the β-Defensin-2 Gene
Source: Genome Biol Evol. 2014 Oct 27;6(11):3025–38. doi: 10.1093/gbe/evu236 (PMC4255768; doi:10.1093/gbe/evu236)
Supplement: Supplementary Data [file supp_evu236_Supplementary_tables.docx]

**Supplementary table 1 β-defensin region copy number calls on 68 human genomic DNA samples**

| **Sample** | **Cohort** | **Population** | **aCGH PC1** | **raw PRT copy number** | **Copy number** | **Batch** |
| --- | --- | --- | --- | --- | --- | --- |
| **CO007** | **HRC** | **UK** | 0.34 | 4.07 | **4** | **1** |
| **CO053** | **HRC** | **UK** | -0.37 | 5.99 | **6** | **1** |
| **CO075** | **HRC** | **UK** | -0.16 | 5.28 | **6** | **2** |
| **CO088** | **HRC** | **UK** | 0.54 | 4.18 | **4** | **2** |
| **CO096** | **HRC** | **UK** | -0.03 | 5.48 | **5** | **1** |
| **CO187** | **HRC** | **UK** | 0.70 | 3.87 | **4** | **2** |
| **CO195** | **HRC** | **UK** | 0.22 | 4.16 | **4** | **1** |
| **CO748** | **HRC** | **UK** | 0.66 | 4.23 | **4** | **2** |
| **CO766** | **HRC** | **UK** | 0.88 | 3.24 | **3** | **1** |
| **CO877** | **HRC** | **UK** | 0.95 | 3.18 | **3** | **1** |
| **CO888** | **HRC** | **UK** | 0.09 | 5.03 | **5** | **1** |
| **CO909** | **HRC** | **UK** | 0.15 | 5.50 | **5** | **2** |
| **CO917** | **HRC** | **UK** | 0.58 | 4.04 | **4** | **2** |
| **CO937** | **HRC** | **UK** | 0.23 | 4.40 | **4** | **2** |
| **NA06991** | **HapMap** | **CEU** | 0.65 | 4.06 | **4** | **4** |
| **NA06993** | **HapMap** | **CEU** | 0.62 | 4.04 | **4** | **4** |
| **NA06994** | **HapMap** | **CEU** | -0.52 | 4.95 | **6** | **3** |
| **NA07000** | **HapMap** | **CEU** | 0.73 | 3.98 | **4** | **4** |
| **NA07019** | **HapMap** | **CEU** | -0.01 | 7.53 | **4** | **1** |
| **NA07022** | **HapMap** | **CEU** | 0.07 | 4.78 | **5** | **4** |
| **NA07029** | **HapMap** | **CEU** | 0.25 | 4.65 | **5** | **2** |
| **NA07034** | **HapMap** | **CEU** | 0.43 | 3.60 | **4** | **5** |
| **NA07048** | **HapMap** | **CEU** | 0.54 | 3.96 | **4** | **5** |
| **NA07055** | **HapMap** | **CEU** | 0.13 | 4.71 | **5** | **5** |
| **NA07056** | **HapMap** | **CEU** | 0.33 | 4.05 | **4** | **4** |
| **NA07345** | **HapMap** | **CEU** | 0.51 | 3.83 | **4** | **5** |
| **NA07348** | **HapMap** | **CEU** | -0.06 | 4.76 | **5** | **4** |
| **NA10830** | **HapMap** | **CEU** | 0.37 | 4.02 | **4** | **2** |
| **NA10831** | **HapMap** | **CEU** | 0.11 | 5.81 | **5** | **2** |
| **NA10835** | **HapMap** | **CEU** | 0.07 | 5.30 | **5** | **4** |
| **NA10838** | **HapMap** | **CEU** | 0.01 | 4.72 | **5** | **2** |
| **NA10839** | **HapMap** | **CEU** | 0.14 | 3.85 | **4** | **2** |
| **NA10846** | **HapMap** | **CEU** | -0.34 | 4.76 | **4** | **1** |
| **NA10847** | **HapMap** | **CEU** | -1.10 | 6.80 | **7** | **1** |
| **NA10851** | **HapMap** | **CEU** | -0.10 | 3.99 | **4** | **1** |
| **NA10854** | **HapMap** | **CEU** | 0.36 | 3.79 | **4** | **5** |
| **NA10860** | **HapMap** | **CEU** | -0.52 | 4.82 | **5** | **1** |
| **NA10861** | **HapMap** | **CEU** | 0.85 | 2.74 | **3** | **4** |
| **NA10863** | **HapMap** | **CEU** | 0.27 | 5.11 | **4** | **5** |
| **NA11831** | **HapMap** | **CEU** | -0.08 | 5.29 | **5** | **5** |
| **NA11832** | **HapMap** | **CEU** | 0.10 | 4.61 | **5** | **5** |
| **NA11840** | **HapMap** | **CEU** | -0.21 | 5.97 | **6** | **5** |
| **NA11995** | **HapMap** | **CEU** | 1.47 | 2.06 | **2** | **2** |
| **NA12005** | **HapMap** | **CEU** | 0.17 | 4.84 | **5** | **5** |
| **NA12144** | **HapMap** | **CEU** | 0.45 | 4.10 | **4** | **5** |
| **NA12146** | **HapMap** | **CEU** | -0.72 | 5.58 | **5** | **1** |
| **NA12154** | **HapMap** | **CEU** | 0.52 | 2.89 | **3** | **5** |
| **NA12234** | **HapMap** | **CEU** | 0.75 | 2.77 | **3** | **5** |
| **NA12239** | **HapMap** | **CEU** | -0.76 | 6.30 | **6** | **2** |
| **NA12249** | **HapMap** | **CEU** | 0.42 | 3.95 | **4** | **5** |
| **NA12707** | **HapMap** | **CEU** | 0.73 | 2.82 | **3** | **4** |
| **NA12716** | **HapMap** | **CEU** | 1.21 | 1.93 | **2** | **2** |
| **NA12752** | **HapMap** | **CEU** | 0.23 | 3.82 | **4** | **2** |
| **NA12760** | **HapMap** | **CEU** | -0.41 | 5.55 | **6** | **3** |
| **NA12801** | **HapMap** | **CEU** | 0.93 | 2.85 | **3** | **2** |
| **NA12802** | **HapMap** | **CEU** | 0.31 | 4.02 | **4** | **2** |
| **NA12864** | **HapMap** | **CEU** | 0.86 | 2.14 | **2** | **1** |
| **NA12865** | **HapMap** | **CEU** | 0.00 | 4.75 | **5** | **2** |
| **NA12873** | **HapMap** | **CEU** | 1.11 | 1.80 | **2** | **3** |
| **NA18500** | **HapMap** | **YRI** | 0.78 | 3.79 | **4** | **5** |
| **NA18502** | **HapMap** | **YRI** | -1.14 | 8.25 | **7** | **3** |
| **NA18562** | **HapMap** | **CHB** | -0.35 | 6.06 | **6** | **5** |

**Supplementary table 2 Position of rhesus macaque orthologues to human β-defensins**

| **Gene (distal to proximal)** | **CHORI-250-65I2** | **CHORI-250-243E20** | **rheMac2 assembly chr8** |
| --- | --- | --- | --- |
| *DEFB107* | Exon 2 112784-112640  Exon 1 108963-108894 | Exon 2 143938-143794  Exon 1 140119-139962 | Exon 2 7989654-7989723  Exon 1 7985878-7986022 |
| *DEFB105* | Exon 1 98665-98545  Exon 2 97277-97236  Exon 3 96896-96827 | Exon 1 129820-129700  Exon 2 128432-128391  Exon 3 128051-127982 | Exon 1 7999959-8000079  Exon 2 8001346-8001387  Exon 3 8001727-8001796 |
| *DEFB106* | Exon 1 94269-94221  Exon 2 91216-91082 | Exon 1 125418-125370  Exon 2 122350-122216 | Exon 1 8004349-8004397  Exon 2 8007523-8007657 |
| *DEFB104* | Exon 1 83921-83864  Exon 2 79295-79134 | Exon 1 115076-115019  Exon 2 110445-110284 | Exon 1 8014809-8014866  Exon 2 8019439-8019600 |
| *SPAG11** | 73139-60312 | 104273-91429 | 8025468-8041135 |
| *DEFB103* | Exon 1 39585-39528  Exon 2 38560-38415 | Exon 1 70703-70646  Exon 2 69678-69533 | Exon 1 8059287-8059344  Exon 2 8060312-8060457 |
| *DEFB2L* (first copy) | Exon 1 28754-28701  Exon 2 26412-26276 | Exon 1 59870-59813  Exon 2 57628-57492 | Exon 1 8070118-8080175  Exon 2 8072369-8072505 |
| *DEFB2L* (second copy) | Exon 1 8507-8450  Exon 2 6667-6531 | Exon 1 39719-39662  Exon 2 37924-37788 | - |

* Multiple splice variants, length of full gene given

**Supplementary table 3 Rhesus macaque BACs identified from library CHORI-250**

| **BAC ID** | **Identification method** | **Sequence ID** | **Genes present, by PCR validation** | **FISH** |
| --- | --- | --- | --- | --- |
| 65I2 | *In silico* and filter hybridization | AC193549.4 | - | No |
| 243E20 | *In silico* and filter hybridization | AC191454.4 | - | No |
| 201P10 | Filter hybridization | Not sequenced | *DEFB2L* | Yes |
| 47B11 | Filter hybridization | Not sequenced | *DEFB2L, DEFB103, SPAG11* | Yes |
| 135L4 | Filter hybridization | Not sequenced | *DEFB2L* | No |
| 148I5 | Filter hybridization | Not sequenced | *DEFB2L* | No |
| 217D13 | Filter hybridization | Not sequenced | *DEFB2L* | No |
| 246K23 | Filter hybridization | Not sequenced | *DEFB2L, DEFB103, SPAG11* | No |
